# Enhancing computation speed and accuracy in deep image prior‐based parameter mapping

**DOI:** 10.1002/mrm.30630

**Published:** 2025-07-10

**Authors:** Max Hellström, Polina Kurtser, Tommy Löfstedt, Anders Garpebring

**Affiliations:** ^1^ Department of Diagnostics and Intervention Umeå University Umeå Sweden; ^2^ Department of Computing Science Umeå University Umeå Sweden

**Keywords:** deep image Prior, denoising, parameter mapping, quantitative MRI, uncertainty estimation

## Abstract

**Purpose:**

To make Deep Image Prior (DIP)‐based parameter mapping faster, more accurate, and suitable for clinical applications, with added support for multislice and 3D datasets.

**Methods:**

DIP leverages the inherent structure of an untrained image generator to address various inverse imaging tasks, including denoising. In this study, we enhance DIP‐based denoising for parameter mapping with warm‐start across neighboring image slices and different patient subjects. This approach leverages spatial similarity to reduce computation time. Additionally, we introduce an early‐stopping criterion that selects the denoising level based on MRI signal noise. We further investigate uncertainty calibration through dropout probability tuning to address issues with miscalibrated uncertainty estimates from Monte Carlo dropout. Furthermore, we explore reducing computation time by tuning learning rates and network complexity.

**Results:**

We show that reusing image generator weights with warm‐start significantly accelerates the denoising of large datasets, reducing computation time by 78% to 95% across various tasks. The early stopping approach proved effective, eliminating the need to manually select the number of optimization steps. Dropout probability tuning helps mitigate the issue of miscalibrated uncertainty, though further refinements are necessary, particularly to achieve better calibration on a per‐pixel level. Additionally, tuning learning rates and network complexity provided valuable insights into optimizing the model for different tasks.

**Conclusion:**

The proposed developments enable DIP‐based parameter mapping to become faster, more accurate, and, consequently, more practical and scalable for clinical applications involving larger datasets.

## 1 INTRODUCTION

MRI contrast is primarily governed by scanner settings (e.g., TR, TE) and intrinsic *tissue parameters* (e.g., T1, T2, ADC). Scanner settings are user‐defined, while tissue parameters reflect the physical and chemical properties of tissue, shaping contrasts like T1‐ or T2‐weighting that reveal distinct anatomical and pathological features.

Quantitative MRI (QMRI) aims to precisely measure tissue parameters using tailored sequences and regression‐based *parameter mapping*.^1^ While this approach enables objective, reproducible measurements, it does not address denoising—allowing noise to propagate through the regression and degrade parameter maps.

To improve the accuracy and precision of parameter mapping, methods generally fall into two categories: *Predefined priors* and *data‐driven methods*. Predefined priors apply explicit denoising constraints, often in image space, such as total variation regularization,^2^, ^3^ Marchenko–Pastur PCA,^4^, ^5^, ^6^ higher‐order SVD,^7^ locally low‐rank models,^8^ and Markov random fields.^9^ Data‐driven methods use machine learning to learn implicit priors, typically via neural networks trained on large datasets. These have been applied to estimating ADC,^10^ relaxation parameters,^11^, ^12^, ^13^, ^14^ and IVIM.^15^, ^16^ Each approach offers specific advantages depending on the application and data availability.

An alternative approach to denoising is Deep Image Prior (DIP),^17^ which does not fall into either of the traditional categories. Unlike predefined priors or data‐driven methods that learn patterns from large datasets, DIP leverages the intrinsic structure of an untrained image generator network. The architecture of the deep learning model itself acts as an implicit prior, enabling denoising without explicitly defined models or external training data.

By optimizing an image generator network solely on the given (noisy) data, DIP effectively captures meaningful patterns, making it a unique solution for different tasks, including denoising. Recent examples of DIP usages in MRI include motion artifact correction of turbo spin‐echo,^18^ real‐time functional cardiac imaging,^19^ and undersampled MRI Reconstruction.^20^

Recently, DIP denoising was integrated into parameter mapping by reinterpreting the weights of an untrained image generator as a parameterization of tissue parameters.^21^ This framework is flexible and adaptable across tasks and incorporates uncertainty via Monte Carlo (MC) dropout.^22^, ^23^, ^24^ Its main limitations are long computation times, manual selection of optimization steps, and calibration issues in MC Dropout uncertainty.

The long computation times pose a significant challenge to clinical implementation, with reported times reaching up to 36min per 2D slice.^21^ This limitation is critical in applications involving large image volumes and study populations. Since each 2D slice is estimated through iterative optimization of an *untrained* image generator, spatial similarities and shared features between slices are overlooked in multislice/3D acquisitions and across study populations. We hypothesize that leveraging these spatial similarities and shared features could substantially reduce the computation time. To address this, we propose investigating *warm‐start* strategies^25^ by reusing image generator weights between adjacent slices and across patients.

Manually selecting the required number of optimization steps is tedious, as it can vary between datasets and even between slices within the same volume. This becomes particularly problematic when computation time needs to be reduced, as prematurely halting the optimization process results in the loss of fine spatial details. To address this, we investigate an objective stopping criterion, based on computing the noise level in the MRI data, thereby minimizing reliance on end‐user subjectivity.

Lastly, MC Dropout uncertainty may be miscalibrated and vary across datasets.^26^ This was also shown for DIP‐based parameter mapping ,^21^ where we attempted post hoc uncertainty recalibration, with limited success. Previous publications conclude that the dropout probability should be adapted to the dataset to achieve well‐calibrated uncertainties,^27^ for example, via grid search.^22^ Several approaches have addressed the topic of calibrating MC Dropout uncertainty, including automatic tuning of the dropout probability,^28^ and various logit scaling methods.^29^ For our use‐case, that is, the combination of DIP, MC Dropout, and parameter mapping, evaluating the performance of tissue parameter uncertainties are, to our knowledge, unexplored, and we aimed to investigate this in more detail by adapting the dropout probability to our data and evaluate this uncertainty on a per‐pixel level.

In this work, we addressed these limitations, with the primary objective of making the DIP method more practical, accurate, and scalable for clinical implementations. Specifically, the goals of this study were to:

- *Reduce computation time* with warm‐start to enable faster denoising of multislice and 3D volumes, making the method suitable for modern imaging demands.
- Improve accuracy and precision by *automatically setting the number of optimization steps* based on the noise level in the MRI signal, eliminating the need for manual tuning.
- *Enhance uncertainty calibration* within the MC Dropout framework, ensuring more reliable uncertainty estimates.

## 2 THEORY

Recently, we proposed a parameter mapping framework that addresses denoising and uncertainty estimation by incorporating DIP and MC Dropout into parameter mapping.^21^ The following section outlines these concepts and suggests how they complement each other in the proposed framework.

### 2.1 Parameter mapping

Let $y\in {\mathbb{R}}^{M\times V}$ be a signal collected through an MRI image acquisition, where a spatial region encompassing V pixels is imaged using M different scanner settings (e.g., flip angles or b‐values). Let $\beta \in {\mathbb{R}}^{P\times V}$ be the underlying tissue parameters to estimate, where P is the number of different tissue parameters estimated in each pixel by a regression. A conventional regression approach to map from measured signal to tissue parameters is a pixel‐by‐pixel approach, that is, for each pixel, v∈{1,…,V}, find the non‐linear least squares (NLLS) estimates such as 

(1)

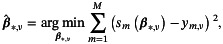


where 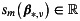 is the signal equation, $y_{m,v}$ is the (m,v)‐th component of y, and $\beta_{*,v}$ denotes all (indexed by ∗) tissue parameters in the v‐th pixel of β. The signal equation is application‐specific, for example, spoiled gradient echo^30^ (SPGR) for Variable Flip Angle (VFA) T1 mapping^31^ or multi‐echo spin echo (MESE) for T2 mapping.^32^ Note that finding the NLLS estimates in Equation (1) corresponds to maximizing a data‐likelihood $p(y|\beta ,\sigma^{2})$ with noise variance $\sigma^{2}$. This is done under the assumption that the model targets are independent and follow a Gaussian distribution, which is a valid approximation in high SNR MRI.^33^

### 2.2 Denoising images with DIP

In DIP‐denoising, a noisy image $x\in {\mathbb{R}}^{V}$ is denoised by utilizing the implicit bias of an image generator. The generator, $f_{\theta }$, a convolutional neural network (CNN) with weights θ, is used to map an input of $N_{c}$ channels of pure noise, $z\in {\mathbb{R}}^{N_{c}\times V}$, to an output image $f_{\theta }(z)\in {\mathbb{R}}^{V}$. The output is interpreted as a *parameterization* of x and denoising is achieved by minimizing the mean squared error (MSE) in the reconstruction,

(2) $$\begin{array}{ll} \hat{\theta } & =\underset{\theta }{\arg\min}\;\text{MSE}\left(f_{\theta }(z),x\right)\\ & =\underset{\theta }{\arg\min}\;\left\{\frac{1}{V}\overset{V}{\underset{v=1}{\sum }}{\left(f_{\theta }{\left(z\right)}_{v}-x_{v}\right)}^{2}\right\}, \end{array}$$

where $f_{\theta }{\left(z\right)}_{v}$ and $x_{v}$ are the v‐th pixel element of $f_{\theta }(z)$ and x, respectively. The denoised image, $\hat{x}$, is the generator output with $\hat{\theta }$ applied, that is, $\hat{x}=f_{\hat{\theta }}(z)$. The minimum of Equation (2) occurs when the generator fully reconstructs x, *including its noise components*. Thus, constraints must be imposed on this minimization to achieve denoising.

### 2.3 Uncertainty from MC dropout

To append the tissue parameter estimate $\hat{\beta }$ with an estimate for its associated uncertainty, $\hat{\sigma }$, we need to modify the generator to enable uncertainty estimation. Gal et al. presented a framework that enables uncertainty estimation by interpreting dropout^34^ in network training as approximate Bayesian inference.^22^ This was later extended to CNNs as approximate Bernoulli variational inference.^23^ With MC Dropout, a deterministic network is redefined as an approximate Bayesian network by adding dropout after each convolution layer. At inference, the conventional forward pass is replaced with $N_{s}$ concurrent forward passes *with dropout still applied* and averaging the results.

### 2.4 DIP‐based parameter mapping

DIP‐based parameter mapping consists of two components. First, DIP is adapted to parameter mapping by modifying the MSE minimization in Equation (2) to map the generator output through the signal equation for all M scanner settings, that is, $s(f_{\theta }(z))\in {\mathbb{R}}^{M\times V}$. This means that we are reinterpreting the generator output as a *parametrization of the tissue parameters* associated with y. Secondly, MC Dropout is enabled by redefining $f_{\theta }$ with the additional input $\epsilon ∼\text{Bern}(p_{d})$, that is, the collection of Bernoulli random variables that are needed to perform MC Dropout with probability $p_{d}$. This combination results in the following minimization problem 

(3)

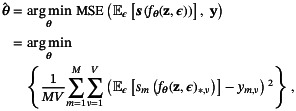




where $f_{\theta }(z,\epsilon )\in {\mathbb{R}}^{P\times V}$ and ${𝔼}_{\epsilon }$ denote the expectation over ϵ. After minimization, the results are estimated using MC Dropout as

(4a) $$\begin{array}{ll} {\hat{\beta }}_{p,v} & =\frac{1}{N_{s}}\overset{N_{s}}{\underset{n=1}{\sum }}f_{\hat{\theta }}{\left(z,\epsilon_{n}\right)}_{p,v}\;\text{and} \end{array}$$

(4b) $$\begin{array}{ll} {\hat{\sigma }}_{p,v} & =\sqrt{\frac{1}{N_{s}}\overset{N_{s}}{\underset{n=1}{\sum }}{\left(f_{\hat{\theta }}{\left(z,\epsilon_{n}\right)}_{p,v}-{\hat{\beta }}_{p,v}\right)}^{2}}, \end{array}$$

∀p∈{1,…,P} and ∀v∈{1,…,V}, where $\hat{\beta }\in {\mathbb{R}}^{P\times V}$ are the tissue parameter estimate, and $\hat{\sigma }\in {\mathbb{R}}^{P\times V}$ its associated uncertainty estimate. From this point onward, we implicitly assume the expectation over ϵ and omit the ${𝔼}_{\epsilon }$ notation for brevity.

The code for the proposed DIP‐based parameter mapping is publicly available on GitHub^1^.

## 3 METHODS

We have identified three key areas for improvement in DIP‐based parameter mapping: Reducing computation time, automatically setting the number of optimization steps, and uncertainty calibration. Section 3.1 outlines the starting point, that is, the previously published framework.^21^ We evaluated several alterations to this framework, each described in detail in Section 3.2.

### 3.1 Baseline method

The baseline method (Algorithm 1) outputs denoised tissue parameter estimates, $\hat{\beta }$, with associated uncertainty, $\hat{\sigma }$, given a signal dataset, y, acquired with MRI signal s. A generator, f, is defined as a five level deep encoder‐decoder type U‐Net^35^ with skip‐connections.^36^ The denoising process begins by defining the generator input and initializing the generator weights. The generator input, $z_{0}$, consists of $N_{c}$ channels of uniformly distributed noise in the range $\left[0,\sigma_{\text{input}}\right]$. The generator weights, $\theta_{0}$, are randomly initialized using PyTorch's default initialization schemes^2^, referred to as InitWeights in Algorithm 1.

The loss function (Equation 3) is minimized by taking $N_{\text{opt}}$ optimization steps with the AdamW ^37^ optimizer with learning rate γ and weight decay λ ^3^. At each step, $z_{0}$ is perturbed using noise‐based regularization with standard deviation $\sigma_{\text{reg}}$, following the method outlined in the original DIP implementation.^17^ Additionally, $\epsilon ∼\text{Bern}(p_{d})$ determines which nodes are randomly dropped (with probability $p_{d}$) to implement MC Dropout. To minimize miscalibration in uncertainty estimates, the dropout probability $p_{d}$ must be selected a priori for each task (see Section 1), e.g., by conducting a grid search over candidate values before parameter mapping with Algorithm 1.

After optimization, MC Dropout samples were collected by conducting $N_{s}$ concurrent forward passes with dropout applied. The estimates for the denoised tissue parameters and their associated uncertainty are then given by Equations (4a) and (4b).

Algorithm 1 was implemented with hyperparameters $N_{s}=1280$, $N_{c}=32$, $p_{d}=0.1$, $\sigma_{\text{input}}=0.1$, $\sigma_{\text{reg}}=0.05$, γ=0.0003, λ=0.0001, and manually tuned $N_{\text{opt}}$ to achieve sufficient denoising for all datasets and applications.

Algorithm 1 **The baseline configuration outputs denoised parameter maps ($\hat{\beta }$) and uncertainties ($\hat{\sigma }$) from input signals (y). Key hyperparameters include optimization steps ($N_{\text{opt}}$), MC Dropout samples ($N_{s}$), and input channels ($N_{c}$). The AdamW optimizer uses learning rate (γ) and weight decay (λ). Dropout is applied with probability $p_{d}$, and noise is controlled via $\sigma_{\text{input}}$ (input) and $\sigma_{\text{reg}}$ (regularization). The signal model s is application‐specific, and $f_{\theta_{0}}$ denotes the untrained image generator.**

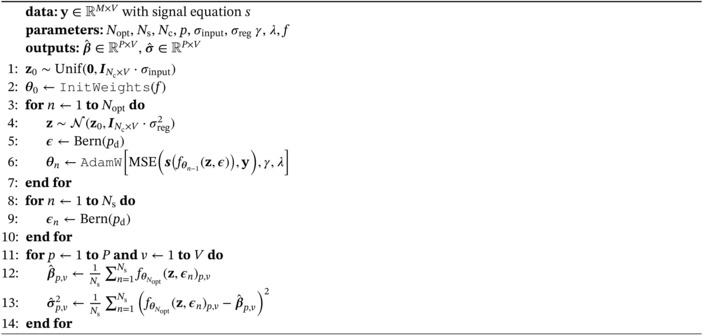

### 3.2 Evaluated alterations

#### 3.2.1 Warm‐start

The warm‐start approach leverages spatial similarities *between adjacent slices* in an imaged volume, as well as *between different image volumes*, that is, between different patient subjects, imaged with the same protocol. By reusing previously obtained generator weights as an initial solution, that is, by modifying line 2 in Algorithm 1, this approach aims to reduce the number of optimization steps required and thus reduce computation time.

**FIGURE 1:**
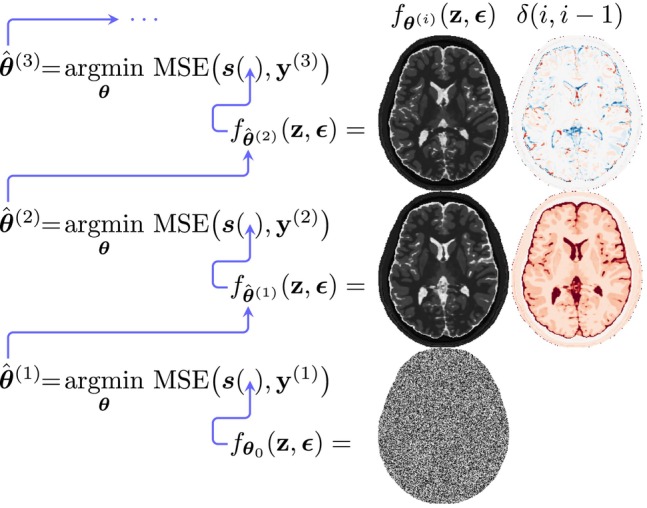
Illustration of DIP‐based parameter mapping with warm‐start. In a multislice/3D volume, the first slice is estimated by optimizing randomly initialized weights $\theta_{0}$. For each subsequent slice (i>1), optimization starts from the previous slice's solution: $\theta_{0}^{\left(i\right)}\leftarrow {\hat{\theta }}^{\left(i-1\right)}$. Based on the assumed spatial similarity between adjacent slices, our hypothesis is that this warm‐start strategy reduces computation time.

Let $y^{\left(1\right)},y^{\left(2\right)},\ldots ,y^{\left(N\right)}$ represent N consecutive 2D slices from a single image volume (single patient) acquired using a multi‐slice or 3D protocol. To enable warm‐start *between adjacent slices*, we estimate the tissue parameters of the first slice with generator weights $\theta_{N_{\text{opt}}}^{\left(1\right)}$ applied according to Algorithm 1. For each subsequent slice, indexed by i∈{2,3,…,N}, we modify the initialization of the weights to

(5) $$\theta_{0}^{\left(i\right)}\leftarrow \theta_{N_{\text{opt}}}^{\left(i-1\right)},$$

which is its optimized state from the preceding slice. This process is illustrated in Figure 1. To enable warm‐start *between different patients subjects*, we transition between volume y and $y^{\prime }$ by initializing the generator weight between transitions to

(6) $$\theta_{0}^{\prime }\leftarrow \theta_{N_{\text{opt}}},$$

which is its optimized state from the preceding patient. The rest of the slices of $y^{\prime }$ are then addressed with Equation (5), that is, warm‐start between adjacent slices.

#### 3.2.2 Early stopping with residual noise estimation

In our previous work,^21^ we used a fixed number of optimization steps ($N_{\text{opt}}$ in Algorithm 1) to minimize Equation (3). This fixed value is manually selected to achieve satisfactory denoising for each individual dataset.^21^ Automatic tuning of $N_{\text{opt}}$ would minimize subjective assessments by the end user. To address this issue, we propose to implement the following changes:

1. Allowing the optimization to terminate with early stopping at a convergence threshold ℓ instead of a fixed $N_{\text{opt}}$.
2. Estimate ℓ from the noise level in the signal dataset.

The first point is easy to implement from MSE since it is calculated during minimization. This is implemented by modifying line 3 in Algorithm 1 to break the optimization loop when MSE≤ℓ. For the second point, we propose to estimate ℓ from the residual standard error.^38^ In parameter mapping, this results in the threshold

(7) $$\begin{array}{l} \ell =\frac{1}{V(M-P)}\overset{M}{\underset{m=1}{\sum }}\overset{V}{\underset{v=1}{\sum }}{\left(s_{m}({\hat{\beta }}_{*,v})-y_{m,v}\right)}^{2}, \end{array}$$

which is the pixel mean of the squared residual standard error, where the $\hat{\beta }$ is computed using a conventional NLLS estimator^4^. This metric reflects the expected noise variance, corrected for degrees of freedom. Stopping when the MSE reaches this level helps avoid overfitting and yields more reliable parameter estimates. Calculating ℓ with Equation (7) is valid for all datasets with M>P scanner settings per pixel.

To assess the impact of warm‐start initialization and early stopping, we performed parameter mapping on a synthetic SPGR patient with known ground truth, enabling NRMSE and SSIM computation.^39^, ^40^ We benchmarked against our baseline,^21^ classical denoisers (NLM,^41^ BM3D^42^, ^43^), and supervised deep learning (DnCNN,^44^ trained on clean–noisy pairs, and Noise2Noise,^45^ using the same architecture but trained with noisy–noisy pairs), representing strong baselines for clean‐supervised and clean‐free learning, respectively. We also tested spatial correlation by comparing warm‐starts from adjacent vs. random slices to simulate large inter‐slice variation. Lastly, we qualitatively evaluated ℓ by mapping parameters at varying scalings and observing effects on denoising and feature visibility.

#### 3.2.3 Uncertainty calibration

With MC Dropout applied, the dropout probability $p_{d}$ determines the probability of dropout and therefore also the shape of the MC sampling distribution. Since $p_{d}$ needs to be adapted to the data at hand,^27^ we performed this adaptation by following the works of Gal et al. with a grid search over possible dropout probabilities.^27^ For this use‐case, we quantified the miscalibration at dropout probability $p_{d}$ as $\hat{\sigma }-\sigma^{\text{ref}}$, that is, the difference between the estimated uncertainty $\hat{\sigma }$ (Equation 4b) and the reference uncertainty $\sigma^{\text{ref}}$. The reference uncertainty was estimated by observing the variation of tissue parameter estimates in $N_{r}$ repeated experiments (identical synthetic data) but with unique noise realizations for each run, that is,

(8) $$\begin{array}{l} \sigma_{p,v}^{\text{ref}}=\sqrt{\frac{1}{N_{r}}\overset{N_{r}}{\underset{n=1}{\sum }}{\left({\hat{\beta }}_{p,v}^{\left(n\right)}-\frac{1}{N_{r}}\overset{N_{r}}{\underset{n^{\prime }=1}{\sum }}{\hat{\beta }}_{p,v}^{\left(n^{\prime }\right)}\right)}^{2}}. \end{array}$$

**FIGURE 2:**
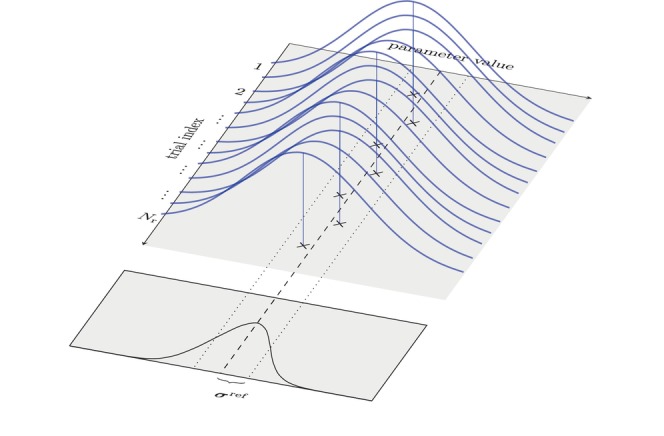
Illustration of miscalibration quantification. With fixed dropout probability $p_{d}$, we simulate $N_{r}$ parameter estimates using identical synthetic data but unique noise realizations to isolate model uncertainty $\sigma^{\text{ref}}$ (Equation 8). The black curve reflects the spread of MC sampling means, while blue curves show trial‐specific uncertainty estimates. Over‐ or underestimation occurs if the blue distributions are consistently wider or narrower than $\sigma^{\text{ref}}$. Calibration is achieved by tuning $p_{d}$ via grid search (Equation 9) to align the distributions.

Equation (8) reflects the expected variation of possible estimates with fixed data, that is, only the noise component varies. For a given dropout probability $p_{d}$, a $\hat{\sigma }-\sigma^{\text{ref}}<0$ reflects that the model underestimates uncertainty, and similarly, $\hat{\sigma }-\sigma^{\text{ref}}>0$ reflects overestimated uncertainty. The latter case is illustrated in Figure 2.

We aimed to find the optimal dropout probability ${\hat{p}}_{d}$, that is, the lowest miscalibration, by performing a grid search over different $p_{d}$ values, with

(9) $$\begin{array}{l} {\hat{p}}_{d}=\underset{p_{d}\in 𝒮}{\arg\min}\;\frac{1}{V}\overset{V}{\underset{v=1}{\sum }}|{\bar{\hat{\sigma }}}_{p,v}-\sigma_{p,v}^{\text{ref}}|, \end{array}$$

where $\bar{\hat{\sigma }}$ is the mean model uncertainty (over $N_{r}$ repetitions with dropout probability $p_{d}$) and 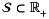 is the search‐window for this grid search. With ${\hat{p}}_{d}$ applied, we analyze the estimated uncertainty on a per‐pixel level to assess whether reliable per‐pixel uncertainty can be achieved in DIP‐based parameter mapping. Since previously chosen $p_{d}=0.10$ resulted in problems with overestimated uncertainty,^21^ we select this value as an upper limit and implement Equation (9) with 𝒮⊂[0.0025,0.10] and $N_{r}=100$. We conducted the grid search on a single slice of synthetic data for T1 and T2 mapping. Slice index 19 (out of 48) was selected for its representative composition of white matter (WM), grey matter (GM), and cerebrospinal fluid (CSF).

#### 3.2.4 Computation time reduction

In addition to evaluating warm‐start, early stopping, and uncertainty calibration, we conducted further investigations aimed at reducing computation time. The total computation time, assuming computation hardware remains the same, is primarily influenced by two key factors: (1) the architecture of the image generator, and (2) its performance when minimizing Equation (3). To reduce computation time, we focus on tuning the most critical parameters associated with these two factors. Specifically, we identified (1) the output filter depth in the convolutions ($N_{f}$), and (2) the learning rate (γ) for the optimizer. For this, we present experiments with one axial slice from one of the in vivo brain VFA patients, see Section 3.3.2 for dataset details.

##### Output filter depth

Altering $N_{f}$ directly impacts the model's capacity, as it determines the number of learnable features. Increasing $N_{f}$ enhances the model's ability to capture complex patterns, but also increases computation time and memory requirements due to the additional storage and processing required for the larger number of feature maps. The baseline method (Section 3.1) used $N_{f}=128$ in all experiments. Here, we investigated whether $N_{f}$ can be tuned to reduce computation time. To test this, we repeated an identical parameter mapping task across a grid of varying $N_{f}$ values, and tracked the computation time at each point. The grid search was conducted with $N_{f}$ ranging from 75 to 500, with early stopping (Equation 7) applied to ensure consistent convergence across all points.

##### Learning Rate

Noting that the prior framework showed slow convergence,^21^ we investigated the optimizer's learning rate (γ in Algorithm 1, line 6). We repeated the tuning process used for $N_{f}$, instead varying γ while tracking computation time.

Larger γ values increase step‐to‐step differences, potentially introducing more variability in parameter estimates at early stopping—a downside of using high γ to reduce computation time. To assess this, we repeated this grid search $N_{r}$ times and computed

(10) $$\sigma_{\gamma }=\frac{1}{V}\overset{V}{\underset{v=1}{\sum }}\sqrt{\frac{1}{N_{r}}\overset{N_{r}}{\underset{n=1}{\sum }}{\left({\hat{\beta }}_{p,v}^{\left(n\right)}-\frac{1}{N_{r}}\overset{N_{r}}{\underset{n^{\prime }=1}{\sum }}{\hat{\beta }}_{p,v}^{\left(n^{\prime }\right)}\right)}^{2}},$$

which is the pixel mean sample standard deviation of estimated tissue parameters when the optimizer operates with learning rate γ.

We conducted this experiment with $N_{r}=50$ and γ ranging from 0.0001 to 0.0039.

### 3.3 Data

The experiments conducted in this study used two different sets of data, synthetic data based on publicly available MRI data of the brain, and in vivo data from MRI‐scans of patients undergoing cancer treatment at the University Hospital of Umeå. The work in this study was conducted in accordance with the principles of the Declaration of Helsinki, with ethical approval (nr. 2016‐220‐31M and nr. 2019‐02666). Informed consent was obtained from all participating patients.

#### 3.3.1 Synthetic data

For the synthetic data, we utilized all 20 available normal anatomical models from BrainWeb^46^, ^47^, ^48^, ^49^, ^50^, ^51^ to generate tissue parameter maps for proton density (PD), T1, and T2. Preprocessing was performed using Hero^5^. For each model, 48 consecutive axial slices with dimensions of 222×185 pixels were selected, and the pixel size was set to $0.98\times 0.98\times 2\;{\text{mm}}^{3}$. These data were used to calculate two sources of synthetic brain scans for parameter mapping: (1) The PD and T1 data were used to calculate synthetic VFA data for T1 mapping with flip angles $\text{FA}\in \{2^{\circ },4^{\circ },11^{\circ },13^{\circ },15^{\circ }\}$ and TR=6.8ms, and (2): The PD and T2 data were used to calculate synthetic multi‐echo SE data for T2 mapping with echo‐times TE∈{50ms,100ms, 150ms}. Proton density was scaled such that the signal spanned the range [0,1] (magnitude normalization). Complex Gaussian noise with σ=0.02 was added to the synthetic MRI signal to simulate visual quality degradation caused by Rice‐distributed noise.

#### 3.3.2 in vivo data

This study utilized two sources of in vivo scans for parameter mapping:

- One VFA dataset for T1 mapping, consisting of brain scans of eight patients (seven male, one female; age span 39–75 years, mean age 52 years):
  Each patient was imaged with a SPGR^30^ sequence (six axial slices of 256×256 pixels, bandwidth 488Hz/pixel). These images were obtained by using the same pixel size, flip angle, TR, and TE as the synthetic VFA data, see Section 3.3.1.
- One DWI dataset for ADC mapping, consisting of prostate scans of 55 patients (age span 45–76, median age 63 years) from the PAMP research project^52^, ^6^).
  As a part of a diagnostic multiparametric protocol, each patient was scanned with diffusion‐weighted EPI acquisitions (b‐values 0, 200, and $1000\;{s/\mathrm{mm}}^{2}$, bandwidth 1953Hz/pixel, and 16 to 20 slices of 80×160 pixels). The pixel data were resampled to remove k‐space zero‐padding. In this study, rather than using all three b‐values, we select only $b=200\;{s/\mathrm{mm}}^{2}$ and $b=1000\;{s/\mathrm{mm}}^{2}$ for ADC mapping^53^ to reduce perfusion components in the ADC maps.

Just as the synthetic data, the in vivo data were also magnitude normalized for each slice. Both in vivo datasets were collected with a PET/MRI scanner (SIGNA PET/MR 3T, GE Healthcare, Milwaukee, WI, USA). To account for variations in field of view and the proportion of anatomic vs. background content, pixels with insufficient signal magnitude—such as those outside the patient volume—were excluded using a binary mask.

## 4 RESULTS

### 4.1 Multi‐slice parameter mapping

**FIGURE 3:**
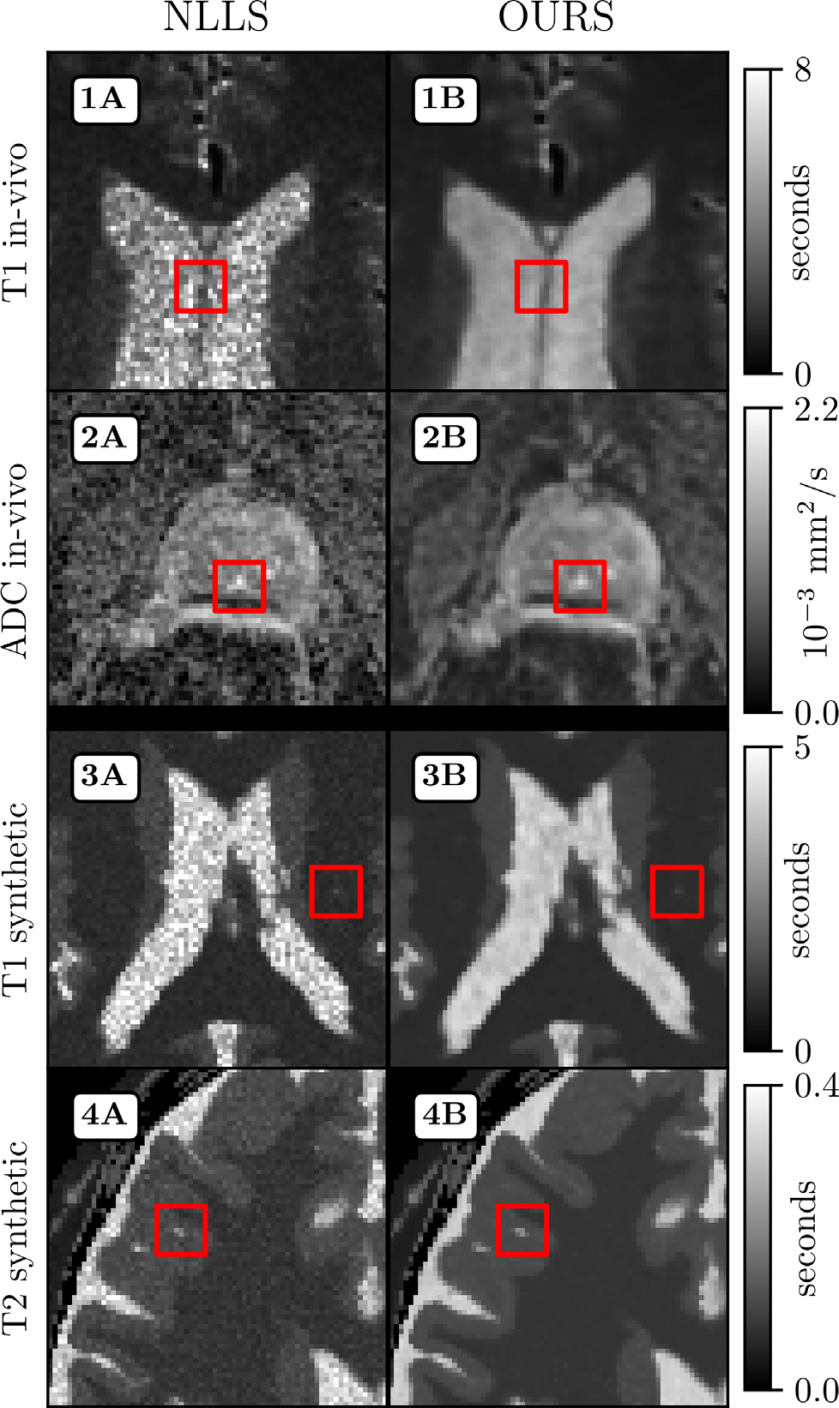
Parameter maps obtained using early stopping and warm‐start (Column **B**) are shown for: T1 in vivo (**1**), ADC in vivo (**2**), T1 synthetic (**3**), and T2 synthetic (**4**), alongside corresponding maps from a conventional NLLS estimator with no denoising (Column **A**). Each map displays a 70×70 pixel region from the central part of the volume. Red markers highlight small anatomical features.

**FIGURE 4:**
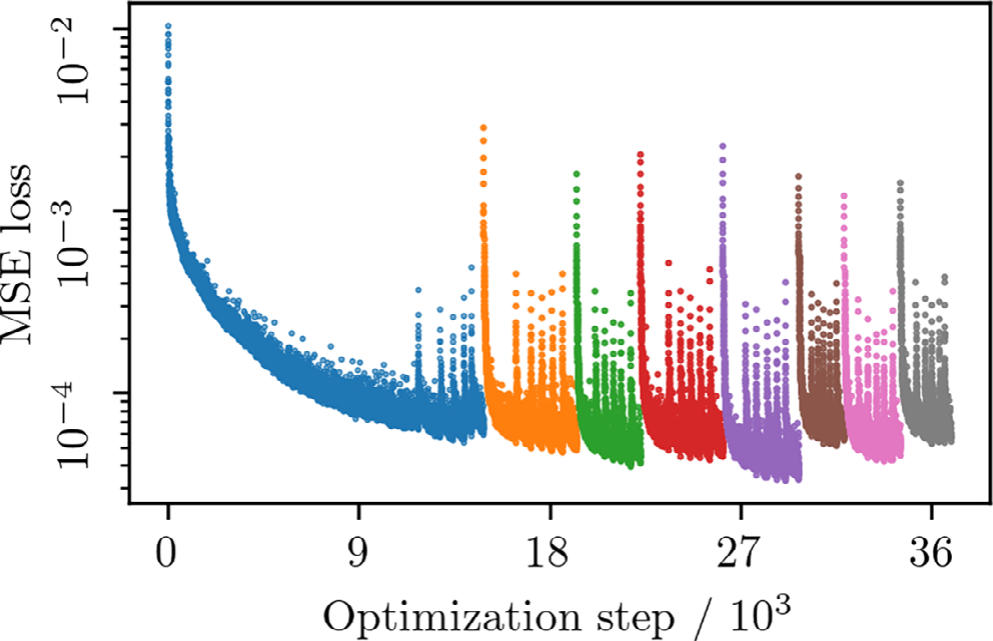
MSE loss with warm‐start. in vivo T1 mapping was performed across eight patients (six axial slices each) using early stopping (Equation 7) and warm‐starting between slices (Equation 5) and patients (Equation 6). The plot shows MSE loss over 36.9 k steps (∼26.3 min), color‐coded by patient. Small loss peaks mark slice transitions; larger peaks mark patient transitions—illustrating effective reuse of generator weights and reduced computation time.

Parameter map volumes were computed for all subjects in this study using the warm‐start approaches outlined in Section 3.2.1. Examples of parameter maps are provided in Figure 3, and the MSE loss curve for the in vivo T1 case is shown in Figure 4. The results in Figure 4 were repeated with each of the dataset's eight patients as the initial subject. This produced a computation time range of 3.9 min (14% relative to the mean) for the entire dataset, with a mean variability of 0.074. (Equation 10).

Comparisons to the baseline method and various reference denoising approaches, followed by experiments with a randomized slice‐ordering scheme, are presented in Appendix A. Details on computation time are presented in Table 1. For all applications but ADC, the parameter mapping optimization was terminated at the limit determined by the early‐stopping criteria found using Equation (7). The ADC case in Figure 3 was optimized to $\text{MSE}=3.1\times 10^{-5}$ (first slice) and a fixed $N_{\text{opt}}=250$ for all concurrent slices. All computations were performed with $N_{s}=128$ and a dropout probability of $p_{d}=0.02$, determined using Equation (9). Compared to estimating a single slice, applying warm‐start reduced the computation time by 78% to 92% per 2D slice when applied between adjacent slices, and by 91% and 95% when also applied between different patients; see Table 1 for details.

**TABLE 1:** The computation time, in minutes, for DIP‐based parameter mapping with and without the warm‐start approaches proposed in Section 3.2.1.

	in vivo data		Synthetic data	
	Brain T1 VFA	Prostate DWI ADC	Brain T1 VFA	Brain T2 MESE
Number of patients	8	55	20	20
Acquisition volume size	6×256×256	(16 to 20) ×80×160	48×222×185	48×222×185
Single‐slice estimation				
Time per slice (min)	7.29±0.71 ^a^	3.76±0.31 ^a^	8.86±0.76 ^a^	10.31±0.81 ^a^
Multi‐slice estimation				
Mean time per slice (min)	1.64±0.18 ^b^	0.39±0.02 ^b^	0.72±0.07 ^b^	1.07±0.06 ^b^
	0.55±0.41 ^c^	0.17±0.03 ^c^	0.54±0.07 ^c^	0.88±0.07 ^c^
Time per volume (min)	9.82±1.07 ^b^	6.23±0.32 ^b^	34.58±3.16 ^b^	51.23±3.09 ^b^
	3.28±2.44 ^c^	2.74±0.52 ^c^	25.92±3.26 ^c^	42.10±3.23 ^c^

*Note*: Single‐slice estimation refers to the time for the first slice in the acquisition volume without warm‐start, while multi‐slice estimation covers the entire volume with warm‐start. Results are reported as mean time per slice and total time for the entire volume. All values are presented as mean ± SD across patients.

^a^ No warm‐start applied.

^b^ Warm‐start applied between adjacent slices.

^c^ Warm‐start applied between adjacent slices and between different patient subjects.

Parameter maps from all included patient subjects (63 in vivo and 20 synthetic), and evaluation of the early‐stopping criteria can be found in the Supporting Information (Figure S1 to S5).

### 4.2 Uncertainty calibration

**FIGURE 5:**
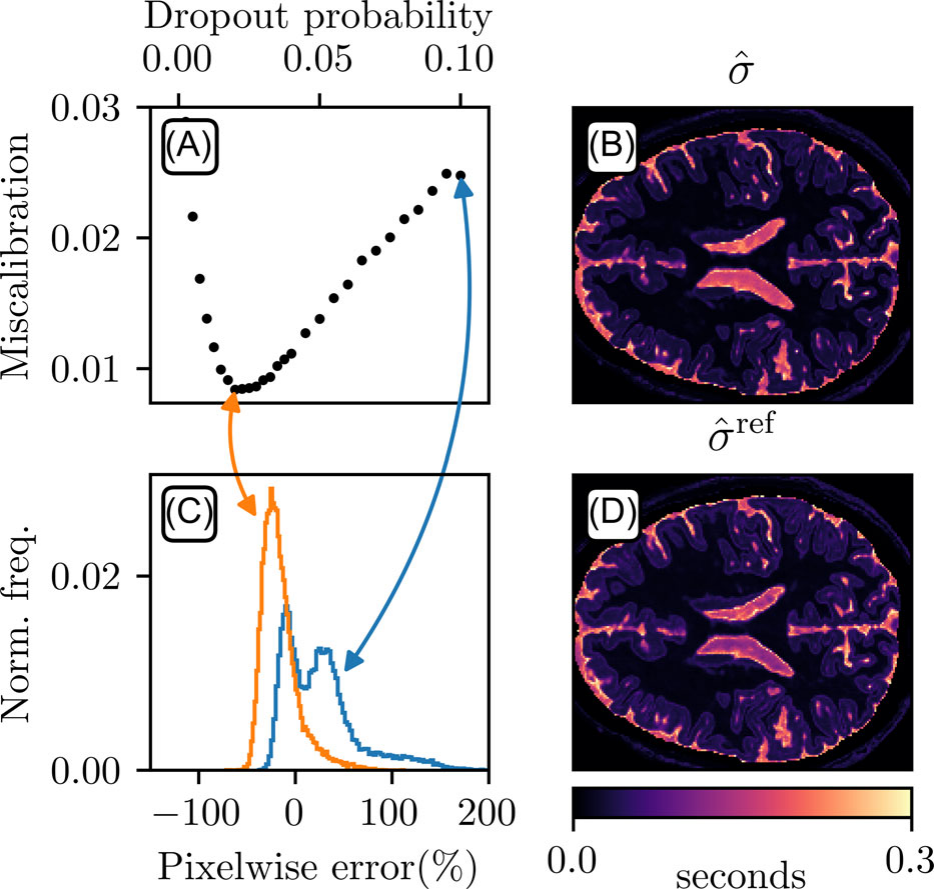
Uncertainty calibration. (A) Miscalibration across dropout probabilities ($p_{d}$), with the optimal value ${\hat{p}}_{d}=0.02$ minimizing miscalibration. With ${\hat{p}}_{d}$ applied, the estimated uncertainty is shown in (B) and the reference uncertainty in (D). (C) Histogram of pixel‐wise miscalibration using ${\hat{p}}_{d}$ (orange) vs. uncalibrated $p_{d}=0.10$ (blue), computed as $(\bar{\hat{\sigma }}-\sigma^{\text{ref}})/\sigma^{\text{ref}}$. Arrows mark each $p_{d}$ and its associated miscalibration. Results show that optimizing $p_{d}$ reduces overall miscalibration but slightly increases underestimation.

The uncertainty calibration, defined by Equation (9), identified an optimal dropout probability of 0.02 for both synthetically generated datasets. Detailed results for the T1 case are shown in Figure 5, with miscalibration across the dropout probability search range 0.0025 to 0.10 (A), histograms of pixel‐wise miscalibration (C), estimated (B) and reference (D) uncertainty.

For T1 mapping, applying the optimal dropout probability reduced the 95th percentile of pixel‐wise miscalibration from 109% to 22% and the 5th percentile from −18% to −39% (Figure 5B). For T2 mapping, the 95th percentile decreased from 102% to 19% and the 5th percentile from −10% to −40%.

### 4.3 Computational efficiency

**FIGURE 6:**
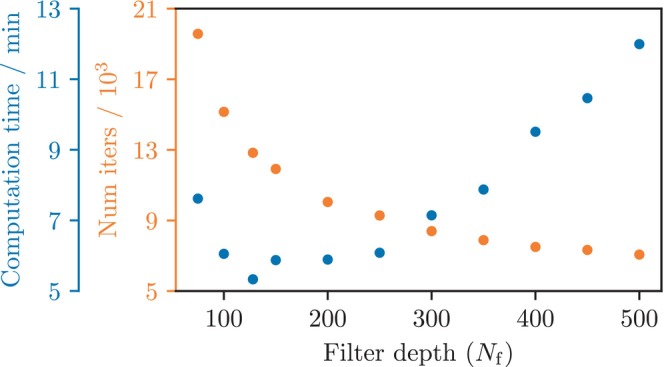
Filter depth grid search. Mean computation time (blue) and optimization steps (orange) for the filter depth grid search. Each point is presented as an average over 15 repetitions. The results indicate that although increasing filter depth reduces the required optimization steps, the corresponding computation time reaches its minimum of 5.3 min with 128 filters applied.

The filter depth grid search (Figure 6) resulted in mean computation times between 5.3min and 12.0min which corresponds to between $7.1\times 10^{3}$ and $19.6\times 10^{3}$ optimization steps. The shortest computation time was achieved with $N_{f}=128$.

**FIGURE 7:**
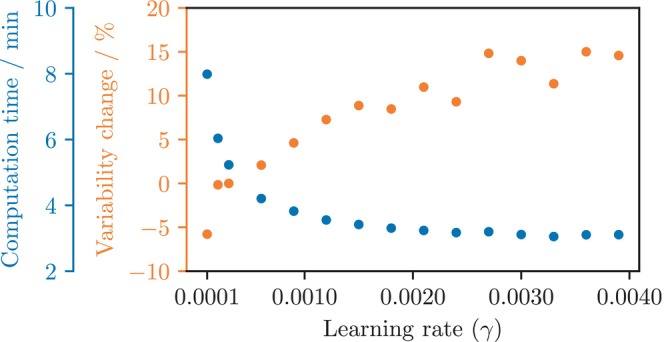
Learning rate grid search. Mean computation time (blue) and variability (Equation 10, orange) across learning rates. Computation time is averaged over 50 runs, and variability is reported relative to the previously used learning rate (γ=0.0003, see Section 3.1). Results show that higher learning rates reduce computation time but increase variability, revealing a trade‐off between speed and model stability.

The learning rate grid search (Figure 7) resulted in mean computation time between 3.1min and 8.0min. The variability metric (Equation 10) varied between −5.8% and +15.0% relative to the baseline γ=0.0003, with the lowest value at γ=0.0001, and the highest value at γ=0.0036.

## 5 DISCUSSION

This paper investigates improvements in DIP‐based parameter mapping,^21^ aiming to make the method clinically viable by reducing computation time and improving reliability. Computation time is reduced via warm‐start and hyperparameter tuning, while reliability is improved through early stopping and uncertainty calibration.

Warm‐start adapts well to this method, enabling estimation of large data volumes with significantly reduced computation time. The baseline method, without warm‐start, makes large‐volume processing impractical due to the time needed to denoise each slice independently.

For instance, the T1 in vivo dataset completed in 26.3 min on a single RTX 2080 Ti GPU. With warm‐start, the mean slice time dropped below one minute across all experiments on the same hardware (Table 1). More broadly, full volumes can now be denoised faster than a single slice previously,^21^ greatly improving applicability—especially on limited hardware.

Early stopping with residual noise estimation adapts well across all tested cases, eliminating the need to manually select the number of optimization steps. This preserves spatial features while producing clean results. Red boxes in Figure 3 highlight preserved fine details, with performance comparable to supervised methods (Section A).

**FIGURE A1:**
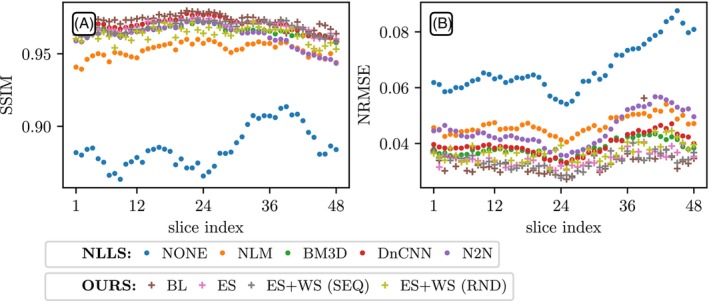
Image quality comparisons. SSIM (**A**) and NRMSE (**B**) scores when applying early stopping (ES) and warm‐start (WS) to DIP‐based parameter mapping. The method performs on par with the supervised approaches, without requiring any external training data. Sequential warm‐starting (SEQ) was 40% faster than randomly initialized warm‐starting (RND), with only minor differences in image quality.

Stopping at MSE values above the threshold underrepresents spatial features, while halting below yields noisy parameter maps (see Figure S5). The warm‐start strategy for multislice or 3D volumes—initializing each slice with its spatial predecessor's weights (Equation 5)—was 40% faster than using random warm‐start slices (Figure A1) and was therefore adopted for all subjects.

Slice order has little impact on image quality (SEQ vs. RND in Figure A1), thanks to early stopping, which limits slice‐to‐slice fluctuations.

Shuffling the patient order in across‐patient warm‐starting (Figure 4) introduced a 3.9‐minute variation in estimation time (14% relative to the mean), likely due to inter‐subject differences in estimated noise levels. However, this had a negligible effect on image quality: The observed variability (0.074, Equation 10) remained below the generator's expected value of 0.095 (γ=0.0003, Figure 7).

We also tested the method in a more challenging scenario: ADC mapping with only two b‐values. Here, Equation (7) can not be employed for early stopping since M>P is not satisfied. Determining noise levels in this DWI data was difficult, possibly due to distortions and artifacts from acquisition and scanner post‐processing. Despite these challenges, empirically determining the early stopping threshold ℓ by monitoring the generator during the first slice, then applying a fixed step count to subsequent slices after warm‐start, proved effective. For example, Figure 3 shows the 8th slice of a 16‐slice ADC volume generated using this approach. Consistent denoising quality suggests the generator weights rapidly adapt to new slices (Equation 5), regardless of patient‐specific variation in concurrent slices. This shows the method's applicability when analytical noise estimation is not feasible.

As described in the literature, miscalibrated uncertainty from MC Dropout sampling is a common problem. For example, Gal et al. ^22^ suggested that grid searching the dropout probability reduces miscalibration. In conventional network‐based approaches, grid search is impractical due to large training datasets. This is not true for DIP‐based methods, as the network trains on just a single data slice. Our findings show that, for our use case, finding a good dropout probability reduces the problem with overestimated uncertainty, but it comes with the price of an increased number of pixels that underestimate the uncertainty (Figure 5). This likely occurs because high‐uncertainty pixels (e.g., CSF in T1 mapping) overly penalize minimization in Equation (9), despite their low proportion. All parameter mapping tasks used the new dropout rate of 0.02, leading to faster convergence than previously reported.^21^ For example, in Figure 4, the first slice converged in 14 870 steps vs. 50 000 in the earlier work. Our results suggest that optimizing dropout, as determined by Equation (9), reduces overall uncertainty and computation time.

However, further investigation is needed, particularly to improve per‐pixel accuracy and test if uncertainty calibration transfers across patients. Future work could explore scaling/binning methods, train with $p_{d}$ as a parameter,^28^ or test alternative sampling for uncertainty estimation.

Tuning dropout and using warm‐start cut computation time, so we also tested learning rate and filter depth. Timing was stable for 100–250 filters; 128 (baseline) was fastest (Figure 6, Section 3.1). Thus, $N_{f}=128$ is a reasonable default, though other setups may benefit from tuning. Learning rate tuning improved convergence (Figure 7) but also raised variability (Equation 10), so increasing it alone isn't effective for faster computation. Adaptive methods may improve speed and stability—e.g., by using a schedule that sharply reduces the step size near the early stopping threshold ℓ.

### 5.1 Limitations and future direction

First, all computations were performed using NVIDIA's parallel computing platform, CUDA^7^, on a single RTX 2080 Ti GPU. While effective, this hardware is now outdated compared to state‐of‐the‐art architectures. Therefore, the usability and efficiency of the proposed methods are expected to improve further with more modern GPUs.

Furthermore, this study focused on brain T1 and T2 mapping, as well as prostate ADC mapping. Future studies could incorporate a broader range of tissue parameters across different body regions to further clarify the applicability of this method.

Finally, in this work, we present results exclusively employing encoder‐decoder U‐Nets as image generators. The use of alternative architectures for parameter mapping remains unexplored and could be investigated in future studies.

### 5.2 Conclusions

We have enhanced DIP‐based parameter mapping^21^ with a focus on increasing its practicality in clinical settings. The key improvements include support for multislice and 3D volumes with warm‐start, and early stopping support reliant on the noise level in the signal data. These alterations have made the method faster, more accurate, and more user‐friendly, substantially enhancing its usability in clinical applications.

## CONFLICT OF INTEREST STATEMENT

Anders Garpebring is a co‐founder of Hero Imaging AB, which develops the analysis software Hero, used to preprocess the data included in this study.

## Supporting information


**Data S1.** Supporting Information.

## Figures and Tables

**FIGURE A2:**
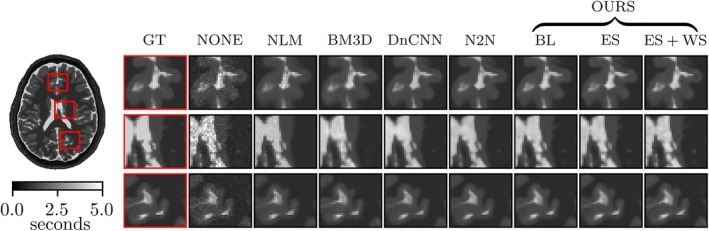
Image quality comparisons. Visual comparison of the results in Figure A1, including the ground truth (GT) and various reference denoising methods. The proposed alterations—early stopping and warm‐start—significantly improve usability, with only a minor impact on image quality.

## ENDNOTES

^1^

https://github.com/MaxHellstrom/dip_parameter_mapping.git.

^2^
The core component of f consists of 2D convolutional layers, which are uniformly initialized. For details, see https://github.com/pytorch/pytorch/blob/main/torch/nn/modules/conv.py.

^3^

https://pytorch.org/docs/stable/generated/torch.optim.AdamW.html.

^4^

Scipy.optimize.least_squares with default method and tolerances, see GitHub repo for full implementation details.

^5^

https://www.heroimaging.com/.

^6^

https://www.umu.se/en/research/projects/pamp‐psma‐acetate‐and‐multiparametric‐mri‐for‐prostate‐cancer‐/.

^7^

https://docs.nvidia.com/cuda/, accessed May 27, 2025.

## APPENDIX A IMAGE QUALITY METRICS AND WARM‐START‐ORDERING

Image quality is presented in terms of SSIM and NRMSE in Figure A1, and visual comparisons in Figure A2. The proposed method (OURS) is presented with the baseline method (BL), and the improved variants with calibrated uncertainty and early stopping (ES) and with the addition of warm‐start, both between adjacent slices (SEQ) and random slice initiation (RND).

As reference methods we applied non‐linear least squares (NLLS) on the raw signal without any denoising (NONE), non‐local means using denoise_nl_means ^8^ with patch_size=7 and patch_distance=11, BM3D via the bm3d package^9^ (noise_type='gw', noise_std=0.02), and two supervised CNN denoisers—DnCNN and Noise2Noise—sharing the same architecture, but trained using clean–noisy and noisy–noisy pairs, respectively, both trained on 19 synthetic VFA patients and evaluated on the twentieth (see supplementary code for details^10^).

## References

[1] Cercignani M , Dowell NG . Tofts PS, eds.Quantitative MRI of the brain. Principles of Physical Measurement. CRC Press; 2018.

[2] Beirinckx Q , Jeurissen B , Nicastro M , et al. Model‐based super‐resolution reconstruction with joint motion estimation for improved quantitative MRI parameter mapping. Comput Med Imaging Graph. 2022;100:102071. doi:10.1016/j.compmedimag.2022.102071 36027768

[3] Löfstedt T , Hellström M , Bylund M , Garpebring A . Bayesian non‐linear regression with spatial priors for noise reduction and error estimation in quantitative MRI with an application in T1 estimation. Phys Med Biol. 2020;65:225036. doi:10.1088/1361-6560/abb9f5 32947277

[4] Grussu F , Battiston M , Veraart J , et al. Multi‐parametric quantitative in vivo spinal cord MRI with unified signal readout and image denoising. NeuroImage. 2020;217:116884. doi:10.1016/j.neuroimage.2020.116884 32360689 PMC7378937

[5] Herthum H , Hetzer S . Tensor denoising of quantitative multi‐parameter mapping. Magn Reson Med. 2024;92:145‐157. doi:10.1002/mrm.30050 38368616

[6] Stern N , Radunsky D , Blumenfeld‐Katzir T , Chechik Y , Solomon C , Ben‐Eliezer N . Mapping of magnetic resonance imaging's transverse relaxation time at low signal‐to‐noise ratio using Bloch simulations and principal component analysis image denoising. NMR Biomed. 2022;35:e4807. doi:10.1002/nbm.4807 35899528 PMC9787782

[7] Zhang X , Xu Z , Jia N , et al. Denoising of 3D magnetic resonance images by using higher‐order singular value decomposition. Med Image Anal. 2015;19:75‐86. doi:10.1016/j.media.2014.08.004 25291148

[8] Shih SF , Tasdelen B , Yagiz E , et al. Improved liver fat and R2* quantification at 0.55 T using locally low‐rank denoising. Magn Reson Med. 2025;93:1348‐1364. doi:10.1002/mrm.30324 39385473 PMC11680733

[9] Baselice F , Ferraioli G , Pascazio V . A Bayesian approach for relaxation times estimation in MRI. Magn Reson Imaging. 2016;34:312‐325. doi:10.1016/j.mri.2015.10.020 26596555

[10] Vasylechko S , Afacan O , Kurugol S . Self Supervised Denoising Diffusion Probabilistic Models for Abdominal DW‐MRI. Comput Diffus MRI. 2023;14328:80‐91. doi:10.1007/978-3-031-47292-3_8 38736559 PMC11086684

[11] Lu Q , Li J , Lian Z , et al. A model‐based MR parameter mapping network robust to substantial variations in acquisition settings. Med Image Anal. 2024;94:103148. doi:10.1016/j.media.2024.103148 38554550

[12] Fu Z , Mandava S , Keerthivasan MB , et al. A multi‐scale residual network for accelerated radial MR parameter mapping. Magn Reson Imaging. 2020;73:152‐162. doi:10.1016/j.mri.2020.08.013 32882339 PMC7580302

[13] Jun Y , Shin H , Eo T , Kim T , Hwang D . Deep model‐based magnetic resonance parameter mapping network (DOPAMINE) for fast T1 mapping using variable flip angle method. Med Image Anal. 2021;70:102017. doi:10.1016/j.media.2021.102017 33721693

[14] Lu Q , Wang C , Lian Z , et al. Cascade of Denoising and Mapping Neural Networks for MRI R2* Relaxometry of Iron‐Loaded Liver. Bioengineering (Basel). 2023;10:209. doi:10.3390/bioengineering10020209 36829703 PMC9952355

[15] Mastropietro A , Procissi D , Scalco E , Rizzo G , Bertolino N . A supervised deep neural network approach with standardized targets for enhanced accuracy of IVIM parameter estimation from multi‐SNR images. NMR Biomed. 2022;35:e4774. doi:10.1002/nbm.4774 35587618 PMC9539583

[16] Huang HM . An unsupervised convolutional neural network method for estimation of intravoxel incoherent motion parameters. Phys Med Biol. 2022;67:215018. doi:10.1088/1361-6560/ac9a1f 36228623

[17] Ulyanov D , Vedaldi A , Lempitsky V . Deep Image Prior. Int J Comput Vis. 2020;128:1867‐1888. doi:10.1007/s11263-020-01303-4

[18] Lee J , Seo H , Lee W , Park H . Unsupervised motion artifact correction of turbo spin‐echo MRI using deep image prior. Magn Reson Med. 2024;92:28‐42. doi:10.1002/mrm.30026 38282279

[19] Hamilton JI , Truesdell W , Galizia M , Burris N , Agarwal P , Seiberlich N . A low‐rank deep image prior reconstruction for free‐breathing ungated spiral functional CMR at 0.55 T and 1.5 T. MAGMA. 2023;36:451‐464. doi:10.1007/s10334-023-01088-w 37043121 PMC11017470

[20] Leynes AP , Deveshwar N , Nagarajan SS , Larson PEZ . Scan‐Specific Self‐Supervised Bayesian Deep Non‐Linear Inversion for Undersampled MRI Reconstruction. IEEE Trans Med Imaging. 2024;43:2358‐2369. doi:10.1109/TMI.2024.3364911 38335079 PMC11197470

[21] Hellström M , Löfstedt T , Garpebring A . Denoising and uncertainty estimation in parameter mapping with approximate Bayesian deep image priors. Magn Reson Med. 2023;90:2557‐2571. doi:10.1002/mrm.29823 37582257

[22] Gal Y , Ghahramani Z . Dropout as a Bayesian Approximation: Representing Model Uncertainty in Deep Learning. arXiv:1506.02142 [stat.ML] 2015.

[23] Gal Y , Ghahramani Z . Bayesian Convolutional Neural Networks with Bernoulli Approximate Variational Inference. arXiv:1506.02158 [stat.ML] 2015.

[24] Kendall A , Gal Y . What Uncertainties Do We Need in Bayesian Deep Learning for Computer Vision? arXiv:1703.04977 [cs.CV] 2015.

[25] Yildirim EA , Wright SJ . Warm‐Start Strategies in Interior‐Point Methods for Linear Programming. SIAM J Optim. 2002;12:782‐810. doi:10.1137/S1052623400369235

[26] Gal Y , Ghahramani Z . Dropout as a Bayesian Approximation: Appendix. arXiv:1506.02157 [stat.ML] 2015.

[27] Gal Y . Uncertainty in Deep Learning. PhD thesis, University of Cambridge. 2016 cs.ox.ac.uk/people/yarin.gal/website/thesis/thesis.pdf

[28] Gal Y , Hron J , Kendall A . Concrete Dropout. 2017 arXiv:1705.07832 [stat.ML].

[29] Laves MH , Ihler S , Kortmann KP , Ortmaier T . Calibration of Model Uncertainty for Dropout Variational Inference. 2020 arXiv:2006.11584 [cs.LG].

[30] Shogry ME , Elster AD . Cerebrovascular Enhancement in Spoiled GRASS (SPGR) Images: Comparison with Spin‐Echo Technique. J Comput Assist Tomogr. 1992;16:48‐53. doi:10.1097/00004728-199201000-00009 1729306

[31] Fram EK , Herfkens RJ , Johnson GA , et al. Rapid calculation of T1 using variable flip angle gradient refocused imaging. Magn Reson Imaging. 1987;5:201‐208. doi:10.1016/0730-725x(87)90021-x 3626789

[32] Woermann FG , Barker GJ , Birnie KD , Meencke HJ , Duncan JS . Regional changes in hippocampal T2 relaxation and volume: a quantitative magnetic resonance imaging study of hippocampal sclerosis. J Neurol Neurosurg Psychiatry. 1998;65:656‐664. doi:10.1136/jnnp.65.5.656 9810933 PMC2170343

[33] Gudbjartsson H , Patz S . The Rician distribution of noisy MRI data. Magn Reson Med. 1995;34:910‐914. doi:10.1002/mrm.1910340618 8598820 PMC2254141

[34] Srivastava N , Hinton G , Krizhevsky A , Sutskever I , Salakhutdinov R . Dropout: A Simple Way to Prevent Neural Networks from Overfitting. J Mach Learn Res. 2014;15:1929‐1958.

[35] Ronneberger O , Fischer P , Brox T . U‐Net: Convolutional Networks for Biomedical Image Segmentation. 2015 arXiv:1505.04597 [cs.CV].

[36] Kaiming H , Xiangyu Z , Shaoqing R , Jian S . Deep Residual Learning for Image Recognition. Comput Vision Pattern Recognit. 2016;770–778. doi:10.1109/CVPR.2016.90

[37] Loshchilov I , Hutter F . Decoupled Weight Decay Regularization. 2017 arXiv:1711.05101v3 [cs.LG].

[38] James G , Witten D , Hastie T , Tibshirani R . An Introduction to Statistical Learning: with Applications in R. Springer Texts in StatisticsNew York: Springer. Springer New York; 2013.

[39] Gonzalez RC , Woods RE . Digital Image Processing. 3rd ed. Pearson; 2008.

[40] Wang Z , Bovik AC , Sheikh HR , Simoncelli EP . Image quality assessment: from error visibility to structural similarity. IEEE Trans Image Process. 2004;13:600‐612. doi:10.1109/tip.2003.819861 15376593

[41] Buades A , Coll B , Morel J‐M . A non‐local algorithm for image denoising. 2005 IEEE Computer Society Conference on Computer Vision and Pattern Recognition (CVPR'05). IEEE; 2005;2:60‐2:65. doi:10.1109/CVPR.2005.38

[42] Dabov K , Foi A , Katkovnik V , Egiazarian K . Image denoising by sparse 3‐D transform‐domain collaborative filtering. IEEE Trans Image Process. 2007;16:2080‐2095. doi:10.1109/tip.2007.901238 17688213

[43] Makinen Y , Azzari L , Foi A . Collaborative Filtering of Correlated Noise: Exact Transform‐Domain Variance for Improved Shrinkage and Patch Matching. IEEE Trans Image Process. 2020;29:8339‐8354. doi:10.1109/TIP.2020.3014721 32784137

[44] Zhang K , Zuo W , Chen Y , Meng D , Zhang L . Beyond a Gaussian Denoiser: Residual Learning of Deep CNN for Image Denoising. IEEE Trans Image Process. 2017;26:3142‐3155. doi:10.1109/TIP.2017.2662206 28166495

[45] Lehtinen J , Munkberg J , Hasselgren J , et al. Noise2Noise: Learning Image Restoration without Clean Data. PMLR; 2018:2965‐2974.

[46] Kwan RKS , Evans AC , Pike GB . An Extensible MRI Simulator for Post‐Processing Evaluation. Lecture Notes in Computer Science. Vol 1131. Springer‐Verlag; 1996:135‐140.

[47] Cocosco CA , Kollokian V , Kwan RKS , Evans AC . BrainWeb: Online Interface to a 3D MRI Simulated Brain Database. NeuroImage. 1997;5:S425.

[48] Collins DL , Zijdenbos AP , Kollokian V , et al. Design and construction of a realistic digital brain phantom. IEEE Trans Med Imaging. 1998;17:463‐468. doi:10.1109/42.712135 9735909

[49] Kwan RK , Evans AC , Pike GB . MRI simulation‐based evaluation of image‐processing and classification methods. IEEE Trans Med Imaging. 1999;18:1085‐1097. doi:10.1109/42.816072 10661326

[50] Aubert‐Broche B , Evans AC , Collins L . A new improved version of the realistic digital brain phantom. NeuroImage. 2006;32:138‐145. doi:10.1016/j.neuroimage.2006.03.052 16750398

[51] Aubert‐Broche B , Griffin M , Pike GB , Evans AC , Collins DL . Twenty new digital brain phantoms for creation of validation image data bases. IEEE Trans Med Imaging. 2006;25:1410‐1416. doi:10.1109/TMI.2006.883453 17117770

[52] Sandgren K , Strandberg SN , Jonsson JH , et al. Histopathology‐validated lesion detection rates of clinically significant prostate cancer with mpMRI, [68Ga]PSMA‐11‐PET and [11C]Acetate‐PET. Nucl Med Commun. 2023;44:997‐1004. doi:10.1097/MNM.0000000000001743 37615497 PMC10566593

[53] Nilsson E , Sandgren K , Grefve J , et al. The grade of individual prostate cancer lesions predicted by magnetic resonance imaging and positron emission tomography. Commun Med (Lond). 2023;3:164. doi:10.1038/s43856-023-00394-7 37945817 PMC10636013

