# Supplementary figures and images for: Enhancing computation speed and accuracy in deep image prior‐based parameter mapping
Source: Magn Reson Med. 2025 Jul 10;94(6):2654–67. doi: 10.1002/mrm.30630 (PMC12501688; doi:10.1002/mrm.30630)

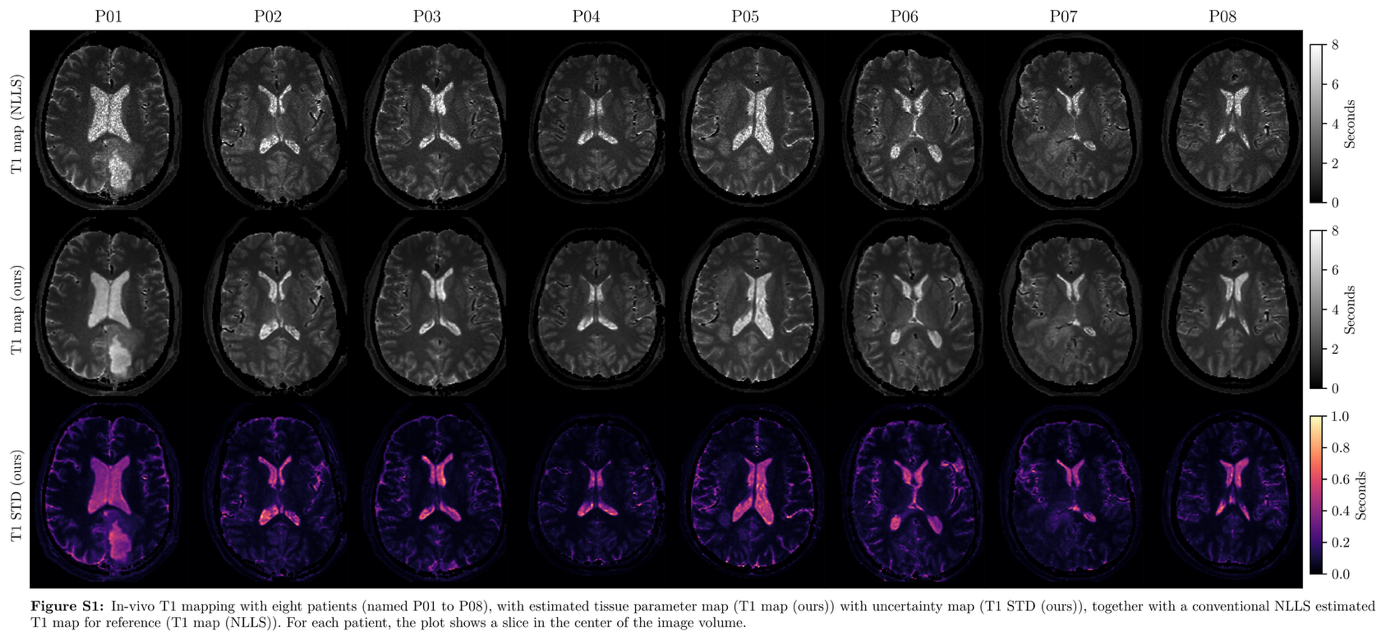

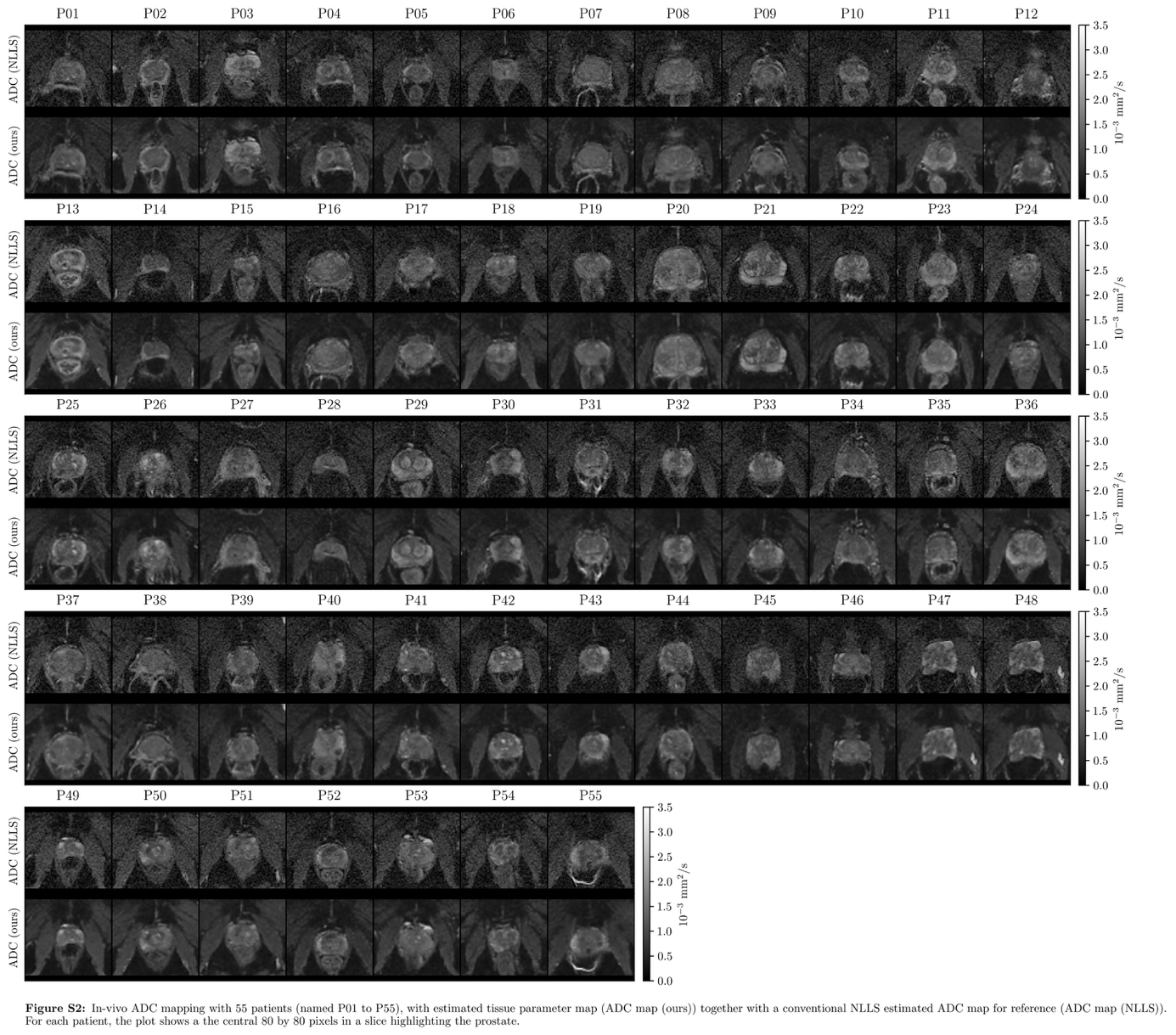


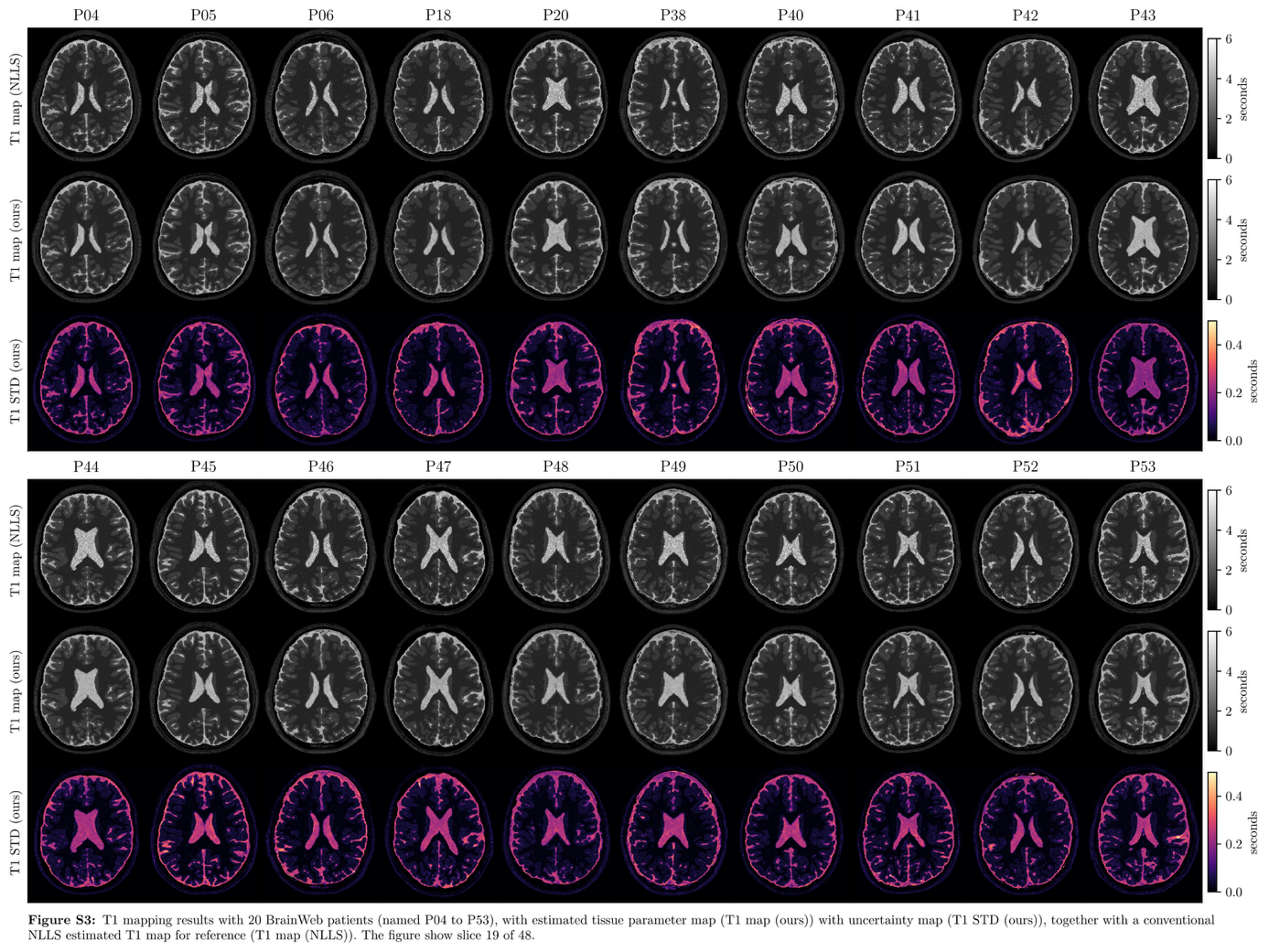


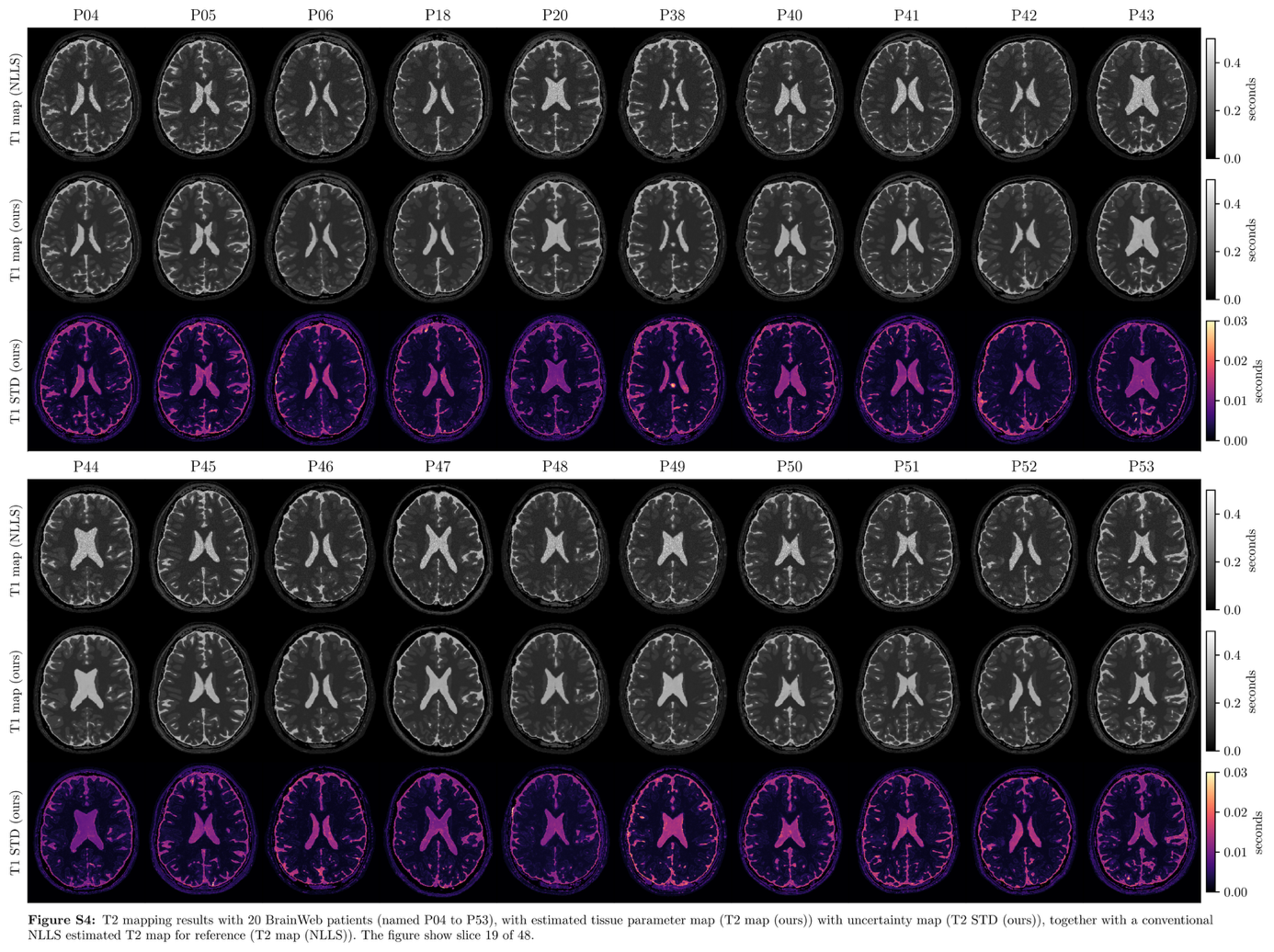


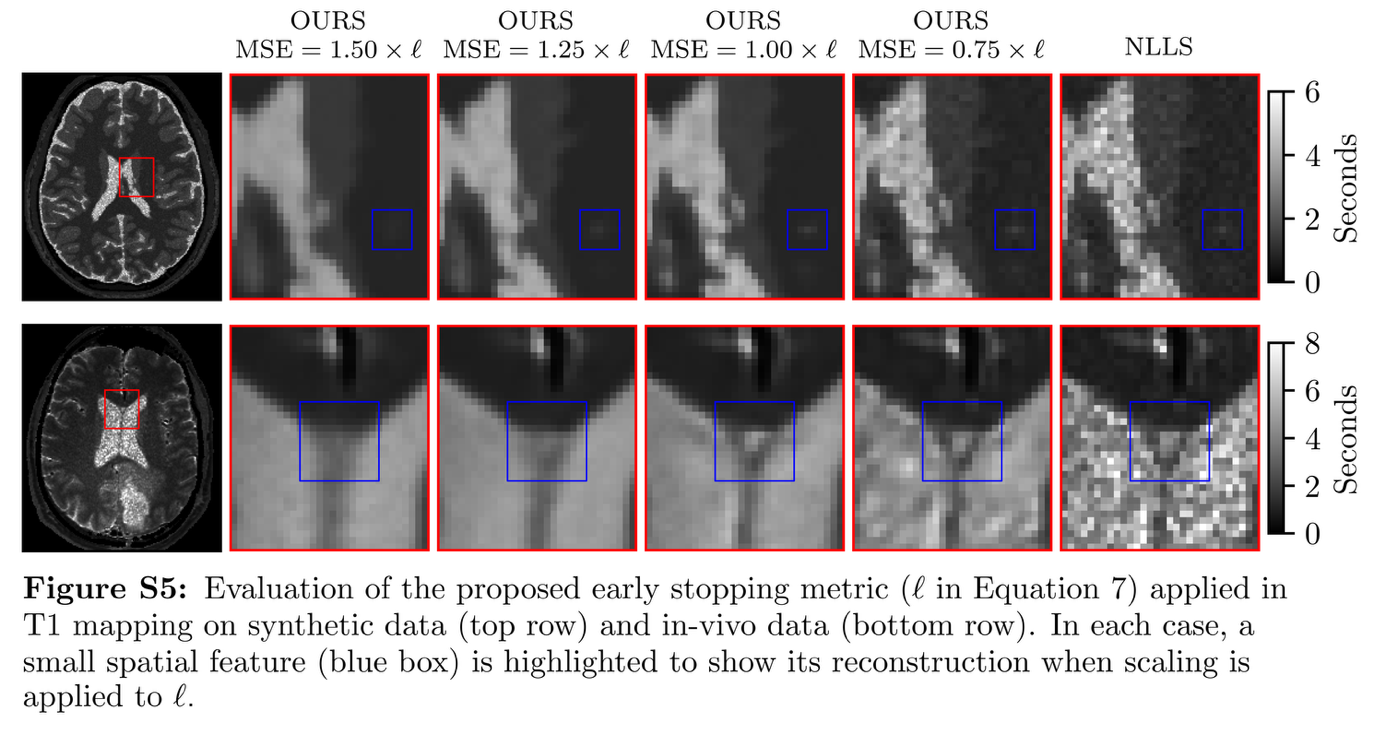

Supplement: Supplementary file 1 — Data S1. Supporting Information. [file MRM-94-2654-s001.docx]
